# Decoding acupoint specificity: from neural patterns to bodily maps

**DOI:** 10.3389/fnins.2026.1815538

**Published:** 2026-04-13

**Authors:** Da-Eun Yoon, Yeonhee Ryu, Younbyoung Chae

**Affiliations:** 1 Department of Meridian and Acupoints, College of Korean Medicine, Kyung Hee University, Seoul, Republic of Korea; 2 Korean Medicine Fundamental Research Division, Korea Institute of Oriental Medicine, Daejeon, Republic of Korea

**Keywords:** acupoint, body maps, clinical practice, indication, neuroimaging, specificity

## 1 Introduction

The question of whether individual acupoints possess intrinsic functional specificity remains one of the most debated issues in contemporary acupuncture research (Choi et al., 2012; Lee D. Y. et al., 2020; Xing et al., 2013). Traditional East Asian Medicine (TEAM) attributes distinct therapeutic actions to specific acupoints, frequently emphasizing not only local but also segmental and general effects (Hwang et al., 2021). Within this framework, acupoints are embedded in a network-like meridian system that links particular body regions and symptom constellations (Jung et al., 2015). In contrast, modern biomedical research has attempted to ground these claims in measurable biological mechanisms, focusing predominantly on local tissue properties, peripheral neuroanatomy, segmental innervation, and central nervous system responses to needle stimulation (Huang et al., 2012; Li et al., 2025). Despite substantial progress in characterizing the sensory, affective, and cognitive dimensions of acupuncture-induced responses, a coherent and widely accepted neurobiological account of point-specific effects has yet to be established (Chae et al., 2013; Kang et al., 2025a). Taken together, TEAM conceptualizes acupoints as nodes within body-wide meridian networks that link distant body regions and symptom constellations, whereas biomedical approaches have largely treated them as localized anatomical sites characterized by their peripheral and central neural encoding.

A major strand of this work has relied heavily on neuroimaging, especially functional magnetic resonance imaging (fMRI), under the assumption that acupoint specificity would manifest as differentiable patterns of brain activation or connectivity (Yan et al., 2005; Zhang et al., 2023). These studies have demonstrated that acupuncture engages distributed cortical and subcortical networks and that multivoxel patterns in somatosensory and higher-order regions can encode information about stimulus location and modality (Jung et al., 2019; Lee I. S. et al., 2020). In this sense, neuroimaging has clarified where and how acupuncture-related inputs are represented in the brain and how richly the central nervous system can discriminate between stimulation sites and modalities (Yoon et al., 2023). However, such findings primarily capture how the central nervous system processes incoming afferent signals—where in the brain a given stimulus is represented and how intensively—not how stimulation at a given point translates into the complex, spatially distributed clinical indications traditionally associated with that point (Kang et al., 2025b; Lee and Chae, 2022). Put differently, the level of description afforded by brain maps does not directly correspond to the level at which acupoint indications are defined, namely, patterns of symptom relief and functional modulation distributed across the body.

In this perspective, we argue that an exclusive reliance on brain-based evidence is conceptually insufficient to fully address acupoint specificity, and we propose a complementary, body-centered mapping framework in which the functional identity of each acupoint is characterized by two empirically derived profiles: sensation maps of evoked bodily sensations and indication maps of clinical symptom regions treated (Jung et al., 2017, 2016).

## 2 Brain-based approaches to acupoint specificity

### 2.1 Evidence from neuroimaging studies of acupoint stimulation

Functional neuroimaging has been widely used to investigate acupoint specificity, typically by asking whether stimulation at different acupoints yields distinguishable patterns of brain activation or connectivity, yet with mixed results regarding the robustness of point-specific effects (Zhang et al., 2023; Rao et al., 2024). In our previous work, we employed both two-point and four-point stimulation paradigms in healthy participants to examine whether different acupoints elicit discriminable multivoxel activation patterns in the brain during noxious needle stimulation (Jung et al., 2019; Lee I. S. et al., 2020). Using multivariate pattern analysis of fMRI data, we found that whole-brain activity could reliably distinguish between stimulation at adjacent body locations, with key contributions from primary somatosensory cortex, motor areas, insula, supramarginal gyrus, and frontoparietal regions that are involved in spatial discrimination of pain. Classification performance was driven by subtle multivoxel differences within largely overlapping networks, rather than by the recruitment of entirely distinct brain regions for each point.

These findings demonstrate that the brain encodes sufficiently rich spatial information to support classification of stimulation sites, even when they are closely adjacent. They also confirm the involvement of distributed somatosensory and higher-order networks in processing acupuncture-evoked pain and *deqi*-like sensations. However, the activation patterns themselves are largely shared across points and mainly reflect generic properties of nociceptive and somatosensory processing, rather than the clinically specific indications attributed to each acupoint. Taken together, these observations highlight both the strength of neuroimaging for decoding where and how acupuncture stimuli are represented in the brain and its limitations in directly capturing the clinically defined notion of point specificity.

### 2.2 Conceptual limits of neuroimaging for explaining point specificity

In this context, the limitations of neuroimaging are not merely technical but, to some extent, intrinsic to what these methods are designed to measure. Techniques such as fMRI are well suited to identifying which brain regions encode the location, intensity, and modality of peripheral inputs, and to detecting differences in activation between conditions or sites (Chae et al., 2013; Kang et al., 2025b). Even when more advanced analyses are applied, such as multivoxel pattern decoding, effective connectivity, or network-level modeling, the primary focus remains on how stimuli and internal states are represented within central circuits. Yet acupoint indications are defined not by where the needle enters the body, but by where and how symptoms are expected to change: patterns of pain relief, modulation of visceral function, or regulation of affective and cognitive states distributed across the organism (Kang et al., 2025a; Lee and Chae, 2021).

Thus, while neuroimaging provides a detailed map of “where and how the brain processes the stimulus,” it does not directly yield a map of the clinical indications associated with a given point. The absence of a straightforward correspondence between brain activation profiles and traditional indications is therefore not merely a technical issue; it reflects a mismatch between the level at which neuroimaging describes central encoding of stimuli and the level at which acupoint specificity is defined—namely, distributed patterns of bodily symptoms and therapeutic effects. Addressing this mismatch requires a framework that operates at the same representational level as clinical indications themselves—namely, spatial patterns of bodily symptoms and therapeutic effects—rather than solely at the level of central stimulus encoding.

Previous studies investigating acupoint specificity have typically focused on differences in peripheral anatomy, segmental innervation, or brain activation patterns following stimulation. While these approaches have provided important insights into the neural encoding of acupuncture stimuli, they rarely address the spatial distribution of clinical effects across the body. The body-centered framework proposed here aims to complement these approaches by examining acupoint specificity at the level where clinical indications are defined: patterns of symptom relief distributed across bodily regions.

## 3 From brain maps to body maps: a conceptual shift

We propose a body-centered mapping framework in which each acupoint is characterized by two spatial profiles: a sensation map of evoked bodily sensations and an indication map of symptom regions treated. By constructing these maps on standardized body templates, acupoint specificity can be compared across points and individuals in a way that aligns more closely with the classical meridian concept than with purely brain-centered models. Crucially, this approach treats the body itself as a measurable representational space in which acupoint identity is defined by reproducible whole-body effect profiles.

### 3.1 Sensation maps: mapping evoked bodily experiences

In this context, sensation maps refer to probabilistic spatial representations of bodily sensations elicited by stimulation of a specific acupoint. These maps capture both local and propagated sensations reported by participants and allow quantitative comparison of spatial sensation patterns across different acupoints. We investigated sensation maps using a bodily sensation mapping (BSM) system, in which participants marked the locations of their sensations on a digital body template after receiving either acupuncture or tactile (von Frey) stimulation at four acupoints (HT7, PC6, ST36, SP10). Individual drawings and *deqi* ratings were aggregated and analyzed statistically to generate probabilistic sensation maps for each point and stimulus type, yielding spatial profiles of propagated and distal sensations (Jung et al., 2016). This procedure transforms subjective reports into pixel-wise probability distributions on a standardized body template, yielding spatial profiles of propagated and distal sensations and enabling quantitative comparison of sensation patterns across acupoints and stimulation modalities.

Acupuncture elicited stronger *deqi* sensations than tactile stimulation, particularly for numbness, spreading, heaviness, and pricking. Unlike tactile input, it also produced sensations that consistently spread to remote body regions. These remote patterns were acupoint-specific: HT7 and PC6 often produced propagated sensations along the medial forearm and upper arm, whereas ST36 and SP10 generated sensations in the abdomen, chest, or along the lower limb, consistent with classical descriptions of “propagated sensation along channels.” Notably, these propagated sensations did not simply mirror dermatomal borders or local receptive field organization, but followed acupoint-dependent trajectories that could be captured as distinct spatial probability maps. Comparing these maps across points provides a data-driven basis for evaluating point specificity at the level of whole-body experiential effects. Within this framework, acupoints can be operationally defined by the similarity or dissimilarity of their sensation maps, allowing clustering into “families” of points that share characteristic patterns of evoked bodily experience.

### 3.2 Indication maps: mapping clinical territories of action

In contrast to sensation maps derived from experimental stimulation, indication maps represent the spatial distribution of clinical symptoms for which a particular acupoint is used in practice. Whereas sensation maps capture acute subjective responses to stimulation, indication maps reflect accumulated clinical knowledge about where in the body an acupoint is used to treat symptoms. Using the same BSM system, we obtained body drawings indicating symptom locations from chronic pain patients in routine practice, together with the acupoints chosen by an experienced clinician, and estimated statistical associations between acupoint selection and the spatial distribution of pain. These relationships were visualized as Z-score maps on a standardized body template, yielding indication maps that highlight regions most strongly associated with each point (Jung et al., 2017). In this way, routine clinical decisions are translated into spatially explicit probability maps that summarize the typical territories of action for each acupoint.

Analysis of commonly used acupoints revealed two main spatial patterns: remote control patterns, as in LI4, ST36, BL40, and BL60, where indications are concentrated in distant regions that often follow the corresponding meridians (e.g., ST36 with the abdomen; BL40/BL60 with the lower back). In contrast, regional control patterns, typically for trunk or back points, showed indications clustering near the point itself. These results are broadly consistent with the clinical view that distal points below the elbows and knees tend to exert remote, meridian-like patterns of clinical indications, whereas many trunk points act more regionally, and they demonstrate that each acupoint has a quantifiable, distinct spatial indication profile. Moreover, because indication maps are defined on a common body template, they can be directly compared across points to assess overlap, distinctiveness, and network-like groupings—for example, by clustering points that share similar territories of symptom relief. This approach provides a concrete, data-driven way to formalize traditional notions of meridian-based relationships and “point families” as measurable patterns of body-wide clinical action.

## 4 Linking bodily maps and brain networks

**Figure 1:**
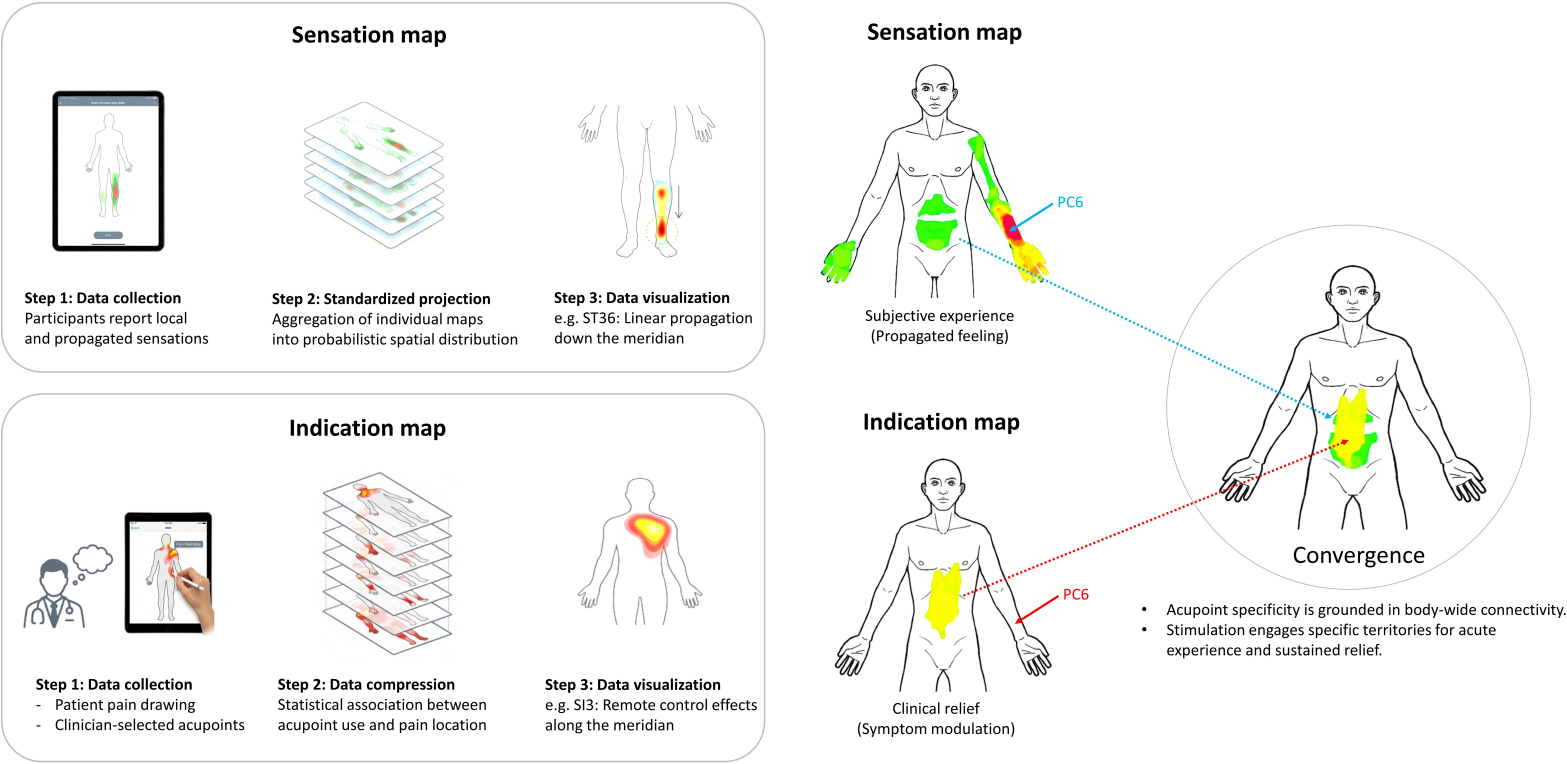
Constructing sensation and indication maps and their convergence at the acupoint level. Left panels: Construction of whole-body maps using a standardized body template. For sensation maps **(top)**, participants draw local and propagated sensations after acupuncture at a given point; these drawings are projected onto a common template and averaged to yield probabilistic maps of evoked sensations (for example, linear propagation from ST36 along the Stomach meridian). For indication maps **(bottom)**, patients draw their pain locations in routine practice and clinicians record the acupoints used; statistical associations between acupoint use and pain locations are then visualized as probability maps of typical symptom regions for each point (for example, remote control effects of SI3 along its meridian). Right panels: Example of an acupoint (PC6) showing sensation maps (subjective propagated feelings) and indication maps (clinical symptom relief) on the same body template. The overlap between these territories (“Convergence”) illustrates how a single point can consistently engage similar body regions at the levels of acute experience and chronic symptom modulation, supporting a body-centered account of acupoint specificity grounded in stable patterns of body-wide connectivity. Example body images were modified from our previous work (Jung et al., 2017, 2016).

A critical test of the body-centered framework is whether sensation maps and indication maps converge. A closely related question is whether these bodily maps can be meaningfully related to central neural representations. Preliminary evidence suggests that, for some acupoints, regions in which participants most frequently experience propagated sensations during experimental stimulation overlap with the body areas for which those points are most commonly used in clinical practice (e.g., PC6 eliciting sensations along the arm and into the abdomen, and being strongly associated with abdominal symptoms). This convergence implies that acupoint specificity may be grounded in stable patterns of body-wide connectivity, whereby stimulation of a given point consistently engages particular distributed territories, both at the level of acute subjective experience and at the level of chronic symptom modulation (Figure 1).

Conceptually, such patterns invite an interpretation in terms of multiscale networks that explicitly link specific peripheral sites to distributed bodily territories and to corresponding large-scale brain systems. To integrate this body-centered perspective with neuroimaging, future studies could (1) simultaneously acquire fMRI data and BSM-based sensation reports during acupoint stimulation, (2) relate inter-individual variability in sensation map extent or topology to corresponding differences in central network engagement, and (3) test whether acupoints with distinct indication maps show correspondingly distinct patterns of functional connectivity in relevant brain networks. For example, one could examine whether participants who report more extensive, meridian-like propagated sensations for a given acupoint also show stronger engagement or coupling within sensorimotor, insular, or default mode networks. In addition, one could test whether acupoints whose indication maps emphasize particular body regions preferentially modulate brain circuits associated with the interoceptive, motor, or affective functions of those regions.

Importantly, the body-centered mapping framework proposed here is not intended to replace the conceptual structure of TEAM, which explains acupoint functions through relationships among meridians, Zang Fu organs, and disease patterns. Rather, the present framework aims to provide an empirically measurable representation of the spatial patterns that may underlie these traditional concepts. In classical theory, the relationships between acupoints and distant symptoms are described in terms of meridian pathways and functional correspondences. In comparison, body-centered maps translate these relationships into quantifiable spatial profiles derived from reported bodily sensations and clinical symptom locations. In this sense, the proposed framework can be viewed as a complementary approach that operationalizes the spatial logic of traditional acupuncture theory in a form that is amenable to empirical analysis and cross-study comparison.

From a clinical perspective, the body-centered mapping framework may offer several practical implications. First, indication maps may help visualize the typical bodily territories associated with individual acupoints, potentially supporting more systematic acupoint selection in clinical practice. Second, comparing spatial profiles across acupoints may allow the identification of clusters of points that share similar bodily effect patterns, which could assist clinicians in selecting alternative points when specific locations are contraindicated or clinically difficult to access. Third, such maps may inform the design of acupuncture clinical trials by enabling more transparent selection of control points that differ in their spatial indication patterns rather than relying solely on anatomical proximity. More broadly, integrating bodily maps with neuroimaging data may help bridge the gap between neural mechanisms and the spatial distribution of clinical effects, thereby contributing to a more integrative understanding of acupoint specificity.

## 5 Conclusion

A shift toward body-centered mapping can provide a more appropriate and empirically tractable framework for examining how individual acupoints exert their characteristic clinical effects. Within this framework, these effects can be understood in terms of each acupoint's whole-body effect profile. By integrating brain-based and body-based representations, future research can characterize multi-level patterns of action that define each acupoint and link acupoint-dependent bodily territories to large-scale brain networks. Acupoint specificity is expressed as reproducible whole-body response patterns that are grounded in both subjective experience and clinical reality. Future studies integrating bodily maps with neuroimaging and clinical outcome measures may help establish testable, spatially explicit hypotheses about acupoint specificity.
